# High-Throughput Phenotypic Screening of Kinase Inhibitors to Identify Drug Targets for Polycystic Kidney Disease

**DOI:** 10.1177/2472555217716056

**Published:** 2017-06-23

**Authors:** Tijmen H. Booij, Hester Bange, Wouter N. Leonhard, Kuan Yan, Michiel Fokkelman, Steven J. Kunnen, Johannes G. Dauwerse, Yu Qin, Bob van de Water, Gerard J. P. van Westen, Dorien J. M. Peters, Leo S. Price

**Affiliations:** 1 Division of Toxicology, Leiden Academic Centre for Drug Research, Leiden, Netherlands; 2 Human Genetics, Leiden University Medical Center, Leiden, Netherlands; 3 OcellO B.V., Leiden, Netherlands; 4 Division of Medicinal Chemistry, Leiden Academic Centre for Drug Research, Leiden, Netherlands

**Keywords:** high-content screening (HCS), high-throughput screening (HTS), 3D culture, polycystic kidney disease, kinase inhibitors

## Abstract

Polycystic kidney disease (PKD) is a prevalent disorder characterized by renal cysts that lead to kidney failure. Various signaling pathways have been targeted to stop disease progression, but most interventions still focus on alleviating PKD-associated symptoms. The mechanistic complexity of the disease, as well as the lack of functional in vitro assays for compound testing, has made drug discovery for PKD challenging. To identify modulators of PKD, *Pkd1*^–/–^ kidney tubule epithelial cells were applied to a scalable and automated 3D cyst culture model for compound screening, followed by phenotypic profiling to determine compound efficacy. We used this screening platform to screen a library of 273 kinase inhibitors to probe various signaling pathways involved in cyst growth. We show that inhibition of several targets, including aurora kinase, CDK, Chk, IGF-1R, Syk, and mTOR, but, surprisingly, not PI3K, prevented forskolin-induced cyst swelling. Additionally, we show that multiparametric phenotypic classification discriminated potentially undesirable (i.e., cytotoxic) compounds from molecules inducing the desired phenotypic change, greatly facilitating hit selection and validation. Our findings show that a pathophysiologically relevant 3D cyst culture model of PKD coupled to phenotypic profiling can be used to identify potentially therapeutic compounds and predict and validate molecular targets for PKD.

## Introduction

Polycystic kidney disease (PKD) is an inherited genetic disorder that is characterized by the formation of renal cysts that block normal tubular function and thereby cause a progressive decline in kidney function with age, typically leading to end-stage renal disease (ESRD) by the sixth decade of life. This prevalent disorder occurs in approximately 1:2500 people^1^ and, in addition to its detrimental effects on kidney function, also affects other organs, such as the liver.^2^

The most prevalent form of PKD, autosomal dominant polycystic kidney disease (ADPKD), is in most cases caused by mutations in the *PKD1* gene or, less commonly, in *PKD2. PKD1* encodes polycystin-1, which is a receptor-like protein thought to be a receptor for various WNT ligands.^3^ *PKD2* encodes polycystin-2, which is a known ion channel with some selectivity for calcium ions. Together, polycystin-1 and polycystin-2 can function as a complex that is thought to be involved in mechanotransduction of urine flow^4^ due to its localization in the cells’ primary cilium.^5^ The polycystin proteins also localize to other areas of the cell, including the plasma membrane^4,6^ and the endoplasmic reticulum (ER).^4^

Loss-of-function mutations in either *PKD1* or *PKD2* or reduced levels of functional protein are causative for cyst formation,^7^ but the mechanisms behind this process are still poorly understood. Dysfunction of the polycystin proteins leads to a reduction in intracellular calcium levels and a consequent rise in intracellular cyclic adenosine monophosphate (cAMP) levels due to the activation of calcium-inhibitable adenylyl cyclase 6 (AC6) and reduced activity of the calcium-dependent cAMP-dependent phosphodiesterases (PDE1/4c).^8^ This increase in cAMP, in turn, leads to alterations in cell proliferation, apoptosis, cell–cell and cell–matrix interactions, and cell polarity.^8^ These events are known contributors to cyst initiation and cyst growth progression.

The mechanistic complexity of this disease has made it particularly difficult to develop effective medicines. As of yet, the only European Medicines Agency (EMA)–approved therapy in Europe for ADPKD is Jinarc (tolvaptan), which is a vasopressin-2 receptor antagonist, consequently requiring the patients to consume large quantities of water due to increase urine production.^9^

In addition to the complexity of the disease, the lack of appropriate in vitro assays to determine drug efficacy is a likely factor underlying the limited choice of therapies available. Traditionally, cells cultured as monolayers have been used to determine drug efficacy and toxicity, but such in vitro systems cannot be used to adequately recapitulate the pathophysiology of ADPKD, since cysts cannot form in a two-dimensional (2D) environment. In contrast, three-dimensional (3D) culture techniques have been developed over the past decade to address these issues and to bridge the gap between 2D monolayers and animal models. Traditionally, these techniques have been generally associated with high costs and low reproducibility and scalability, but due to their physiological relevance, 3D phenotypic screening techniques have become a fundamental research tool in many fields,^10^ including cancer research.^11^

In order to identify effective molecules and therapeutic targets in a more physiologically relevant model, we have developed a high-content and high-throughput screening platform that uses 3D-cultured cysts and used this to screen a kinase inhibitor library with known molecular targets. This allowed us to relate compound efficacy to molecular targets potentially involved in cyst growth.

## Materials and Methods

### Generation and Cloning of Cell Lines

To generate cells with reduced *Pkd1* gene expression, wild-type mouse inner medullary collecting duct (mIMCD3, ATCC CRL-2123) cells were transduced with a lentivirus containing a short hairpin against *Pkd1*. Lentiviral constructs expressing shRNAs targeting *Pkd1* (TRCN0000072084, 085, 086, and 087) and a nontargeting control construct (SHC002) were obtained from the Sigma MISSION shRNA library (Sigma-Aldrich, Zwijndrecht, Netherlands). Production of lentiviruses by transfection into 293T cells has been described earlier.^12^ Cells were selected using puromycin. Reduced *Pkd1* expression, approximately 60%, was confirmed by qPCR (**Suppl. Fig. S1A**, mIMCD3 sh*Pkd1*), and a cell line transduced with construct TRCN0000072084 was selected for our studies. This cell line was used for the majority of the experiments described here. In addition, *Pkd1* knockout mIMCD3 cell lines were generated (**Suppl. Fig. S2**) using the dimeric CRISPR RNA-guided FokI nuclease (RFN) method^13^ in mIMCD3 cells. In short, the RFNs for *Pkd1* exon 15 were selected using ZiFiT (http://zifit.partners.org/ZiFiT/Disclaimer.aspx) and cloned into vector pSQT1313neo as described previously (http://zifit.partners.org/ZiFiT/Program_use.aspx#_CRISPR_RFNs) (**Suppl. Table S1**). In the pSQT1313neo construct, we replaced the ampicillin gene of pSQT1313 with the kanamycin/neomycin resistance cassette of pEGFP-N1 (Clontech, Mountain View, CA) to facilitate G418 selection of clones that have taken up pSQT1313neoRFN and enrich for clones that carry a *Pkd1* exon 15 deletion (pSQT1313 obtained from Addgene, Cambridge, MA). One clone with the correct sequence was selected and cotransfected with pSQT1601 (Addgene), the plasmid expressing the Csy4 and dCas9-FokI fusion proteins. mIMCD3 cells were grown to 80% confluency in a 9 cm petri dish and transfected with 2 µg of Pkd1ex15RFN and 8 µg of pSQT1601 DNA using Lipofectamin 2000 (Invitrogen, Waltham, MA). The G418 (0.5 mg/mL) selection was applied after 48 h. After 7 days, cells were replated at a density of ~50 cells per 9 cm plate. Single colonies were picked and analyzed using PCR with primers flanking the RFN target sites (**Suppl. Table S2**). PCR products were digested with restriction endonuclease *Pvu*II, which cuts between the Pkd1ex15RFN target sites. From clones that showed undigested PCR products, demonstrating a deletion of the *Pvu*II restriction site on both alleles, the PCR products were subcloned using the TOPO cloning kit (Invitrogen). Fifteen subclones were analyzed by Sanger sequencing. The sequences for clone 5E4 revealed a 25 bp out-of-frame deletion on one allele and an 295 bp out-of-frame deletion for the other allele. These cells are denoted as mIMRFNPKD 5E4 throughout this publication.

### Maintenance of Cell Lines

All mIMCD3 cell lines were cultured in 175 cm^2^ culture flasks at 37 °C in an environment of 5% CO_2_ in DMEM/F12 (Ham’s) culture medium (D8062, Sigma-Aldrich), supplemented with 10% fetal bovine serum (FBS, Gibco Fisher Scientific, Landsmeer, Netherlands), Glutamax, and penicillin/streptomycin. Before maximal cell density was reached, cells were washed with 1× phosphate-buffered saline (PBS) (Sigma-Aldrich) and trypsinized with 1× trypsin (Gibco Fisher Scientific). Medium was subsequently added and cells were pelleted by centrifugation for 5 min at 1500 rpm. The cell pellet was resuspended in FBS with 10% DMSO (Biosolve B.V., Valkenswaard, Netherlands) and frozen in aliquots to −150 °C.

### 3D Cyst Assay

**Figure 1.**
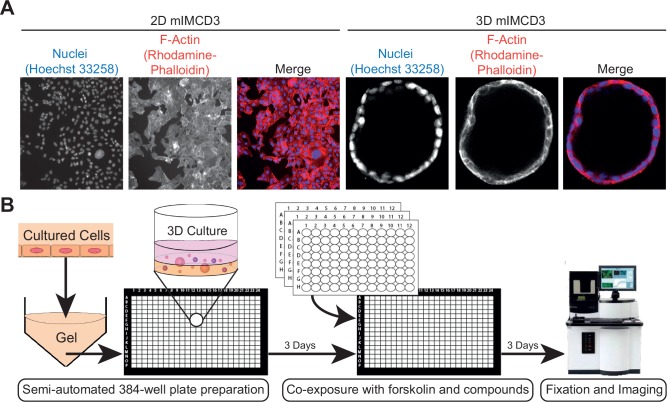
3D high-content screening platform that uses mIMCD3 cysts grown in hydrogels as an in vitro model for PKD. (**A**) mIMCD3 sh*Pkd1* cells grown on culture plastic cannot recapitulate cystic structures (left panel); in contrast, mIMCD3 sh*Pkd1* cells can form cysts when grown in a 3D microenvironment (right panel). (**B**) 3D high-content screening platform that uses mIMCD3 sh*Pkd1* or mIMRFNPKD 5E4 cysts to determine compound efficacy.

For the primary screen of the SelleckChem library (Munich, Germany), cryopreserved mIMCD3 cells were quick-thawed using a 37 °C water bath and immediately added to Cyst-Gel (OcellO B.V., Leiden, Netherlands). For all subsequent experiments, including the validation screen, we modified this procedure to allow cryopreserved cells to recover in a 2D culture for 72 h prior to use in the 3D assay, which resulted in improved cyst homogeneity and overall assay performance. Therefore, for all subsequent experiments, cryopreserved mIMCD3 cells were quick-thawed in a 37 °C water bath and added to 175 cm^2^ culture flasks and cultured for 72 h at 37 °C in an environment of 5% CO_2_ prior to initiation of the cyst assay. After 24 h, medium was refreshed, and after 72 h, the monolayer was washed with 1× PBS (Sigma-Aldrich) and cells were trypsinized using 1× trypsin (Gibco Fisher Scientific) and mixed with Cyst-Gel (OcellO B.V.). A cell–gel mix (14.5 µL) was pipetted to 384-well plates (Greiner µClear, Greiner Bio-One B.V., Alphen aan den Rijn, Netherlands) using a CyBi Selma 96/60 robotic liquid dispenser (Analytik Jena AG, Jena, Germany). The gel–cell mix was plated at a final cell density of 2175 cells per well. After gel polymerization at 37 °C for 30 min, 33.5 µL of medium was added to each well. Cells were grown in gel for 72 h (in order to initiate lumen formation prior to compound exposures), after which the cells were coexposed with forskolin (*Coleus Forskohlii*, Calbiochem, Millipore BV, Amsterdam, Netherlands) and molecules to be tested alongside positive controls using the CyBi Selma 96/60 or a BioMek FXP (Beckman Coulter B.V., Woerden, Netherlands). After 72 h, cultures were fixed with 4% formaldehyde (Sigma-Aldrich) and simultaneously permeabilized with 0.2% Triton-X100 (Sigma-Aldrich) and stained with 0.25 µM rhodamine-phalloidin (Sigma-Aldrich) and 0.1% Hoechst 33258 (Sigma-Aldrich) in 1× PBS for 12 h at 4 °C, protected from light. After fixation and staining, plates were washed in 1× PBS for 12–24 h, after which plates were sealed with a Greiner SilverSeal (Greiner Bio-One B.V.) and stored at 4 °C prior to imaging (screening procedure illustrated in **Fig. 1**).

### Compounds

A kinase inhibitor library containing 273 compounds (L1200) was obtained from SelleckChem (Munich, Germany), with compounds predissolved to 10 mM in DMSO. Analytical-grade DMSO was obtained from Biosolve B.V. Rapamycin, roscovitine, sorafenib tosylate, torin 1, and buparlisib (NVP-BKM-120) were purchased from SelleckChem through distributor Bio-Connect B.V. (Huissen, Netherlands). Metformin HCl was obtained from Sigma-Aldrich.

### Fluorescence Microscopy

Hoechst 33258 and rhodamine-phalloidin–stained cysts in 384-well plates were imaged using a BD Pathway 855 (BD Biosciences, Breda, Netherlands) automated inverted wide-field microscope using a 4× Olympus objective. Images were obtained using BD Attovision software (BD Biosciences) accompanying the microscope, which was used to image focal planes throughout the gel at intervals of 50 µm. The gel was imaged through its entire depth (*z* axis), requiring around 25 images per well; each image captured approximately 75% of the well area (**Suppl. Fig. S3A**). High-resolution confocal images were made using a 20× objective on a Nikon Ti Eclipse confocal laser microscope (561 and 408 nm lasers). Confocal images were exported using NIS Elements Viewer (Nikon Instruments Europe B.V., Amsterdam, Netherlands).

### Image and Data Analysis

Images obtained with the BD Pathway 855 imager were processed through Ominer software (OcellO B.V.) integrated in the KNIME Analytics Platform (Konstanz, Germany, http://www.knime.org/). This software allowed the quantification of images derived from both rhodamine-phalloidin (F-actin) and Hoechst 33258 (nuclei) image channels. This procedure is schematically represented in **Supplemental Figure S3B**. For the rhodamine-phalloidin image channel, a monochrome mask was generated for all the individual images taken along the *z* axis of the gel to define regions of interest (ROIs). This approach allowed us to quantify individual cysts—even those vertically overlapping that would otherwise have been detected as one object. These monochrome masks were used to derive shape- and size-related phenotypic descriptors of each cyst. Cyst size was derived from these monochrome masks as an area measurement of the number of pixels occupied per cyst (with approximately 100–150 cysts in each well, **Suppl. Fig. S4**). The actin-dense region at the boundary of all cysts was quantified separately, so that the thickness and shape of the cyst wall could be related to the total cyst size. Finally, to derive staining intensity-related quantifications, a maximum intensity projection of the original image stack was generated. For the Hoechst 33258 image channel, the spatial resolution upon imaging with the BD Pathway 855 was not high enough to accurately measure the shape of individual nuclei. Therefore, nuclei image stacks were immediately processed to a maximum intensity projection and processed as described earlier.^14,15^ All phenotypic descriptors were subsequently *z* score normalized to plate medians and thereafter *z*-scored to unstimulated control median or scaled to percent inhibition (unstimulated control median to 100%, forskolin-stimulated control median to 0%) using KNIME. For multiparametric phenotypic analysis, phenotypic descriptors were ranked based on Z-prime values of less than −1.0 between stimulated (2.5 µM forskolin) and 10 nM rapamycin-treated, forskolin-exposed control conditions for all cysts. **Supplemental Table S3** provides an overview of included phenotypic features. Selected phenotypic features were used for principal components analysis (PCA) to condense phenotypic information to three principal components (PC0, PC1, and PC2), together comprising 84% of the variation in the dataset. Principal components were exported from KNIME and visualized as a 3D scatterplot generated using the Scatterplot3D package (https://CRAN.R-project.org/package=scatterplot3d) for RStudio 0.99.878 (https://www.rstudio.com/products/rstudio2) with R3.2.3 (https://www.r-project.org/). Other charts were made using the ggplot2 (http://ggplot2.org) package for RStudio 0.99.878 with R3.2.3 or with GraphPad Prism 7 (GraphPad Software, La Jolla, CA).

### Assessment of Synergy

The Bliss independence model^16^ was used to assess potential synergistic effects. This model assumes that the effects of a certain drug are independent of those of the other drug. To apply this method, inhibition of cyst growth was scaled between 0 (no inhibition) and 1 (maximum effect). The model is given in the formula *E* = *E_A_* + *E_B_ – E_A_E_B_*, where *E* is the predicted combined compound response based on the individual effect of two compounds (*E_A_*, torin 1; *E_B_*, buparlisib). This method compares the observed combination response with the predicted combined compound response *E* in a combination index (*CI* = (*E_A_* + *E_B_ – E_A_E_B_*)/*E_AB_*). The combination is declared synergistic if the observed effect is larger than the predicted combined response, or antagonistic if the observed response is smaller than the predicted response.

### Statistical Calculations

Unless otherwise stated in figure legends, figures represent mean and standard deviations (SDs). All statistical calculations were performed in GraphPad Prism 7. Z′-factor calculations were performed after correction for the number of replicates to reflect assay robustness:^17^

$$Z^{\prime }factor=\frac{\left(AVG_{max}-\frac{3SD_{max}}{\sqrt{n}}\right)-\left(AVG_{min}+\frac{3SD_{min}}{\sqrt{n}}\right)}{AVG_{max}-AVG_{min}}\;(\mathrm{eq}1{)}^{17}$$

In eq 1, *AVG* is the mean and *SD* the standard deviation of stimulated (*max*) or unstimulated (*min*) control groups, and *n* is the number of technical replicates.

## Results

### mIMCD3 Cells Form Cysts in 3D Hydrogels and Addition of Forskolin-Induced Cyst Swelling

**Figure 2.**
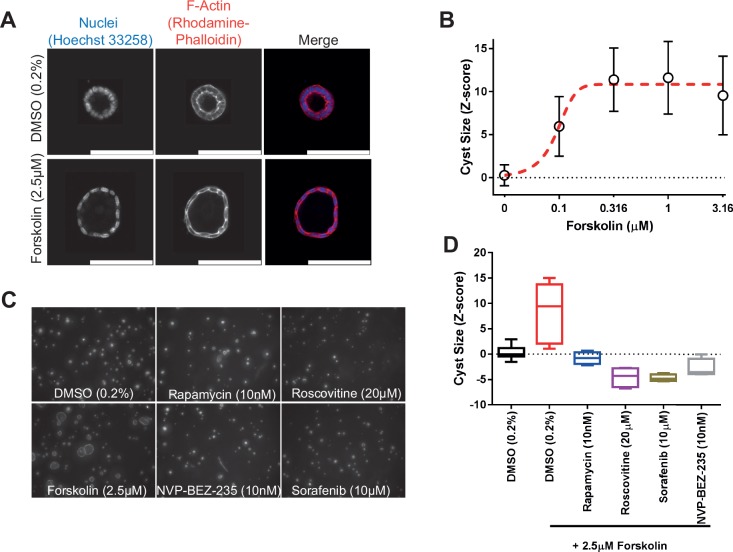
mIMCD3 sh*Pkd1* cyst growth can be enhanced with forskolin, and this increase can be prevented with positive control compounds rapamycin, sorafenib, roscovitine, and NVP-BEZ-235. (**A**) High-resolution images obtained from an unexposed (0.2% DMSO, top panel) and a forskolin-treated (bottom panel) cyst. (**B**) Using Ominer analysis tools, cyst size could be expressed as a function of forskolin concentration. Data points represent means ± SD of eight replicate wells (technical replicates). (**C**) Representative images of control compounds after coexposure with forskolin, including unstimulated (0.2% DMSO) control. (**D**) Quantification of average cyst size from C. Whiskers represent min to max. Rapamycin, roscovitine, sorafenib, and NVP-BEZ-235 tested in quadruplicate (technical replicates), unstimulated (*n* = 16 replicate wells) and stimulated (*n* = 8 replicate wells). These experiments have been performed independently more than three times (**Suppl. Figs. S1B,C** and **S5A,C** also show the performance of control molecules in biological replicates).

To identify modulators of cyst growth, we developed an automation-compatible 3D cyst growth assay, in which cyst swelling is driven by forskolin over a period of 72 h. mIMCD3 cells with a short-hairpin-mediated knockdown of *Pkd1* were cultured in extracellular matrix–based hydrogels in 384-well plates and formed cystic structures by day 3, whereas cysts could not form in 2D monolayer cultures (**Fig. 1A**). Cysts were exposed to solvent (0.2% DMSO, unstimulated) in culture medium or 2.5 µM forskolin (stimulated) and responded to exposure to forskolin by increased cyst swelling (**Figs. 1B** and **2A**). Cyst size was measured by quantifying object area from all individual cysts. The mean cyst area and standard deviation of eight replicates is shown as a function of forskolin concentration and presented as the *z* score relative to the unstimulated control median (**Fig. 2B**). It should be noted that this forskolin-driven cyst growth is an intrinsic characteristic of mIMCD3 cells, and is not dependent on the presence of polycystin-1 (**Suppl. Fig. S1B,C**). Coexposure with forskolin and rapamycin (inhibitor of mammalian target of rapamycin [mTOR]), roscovitine (inhibitor of cyclin-dependent kinases: Cdc2, CDK2, and CDK5^18^), NVP-BEZ-235 (a dual inhibitor of mTOR/PI3K^19^ that potentially also inhibits ATM and ATR at high concentrations^20^), or sorafenib (sorafenib tosylate, a multikinase inhibitor targeting B-Raf and Raf-1^21^) prevented the increase of cyst swelling induced by forskolin (**Fig. 2C**,**D**). The increase in cyst growth by forskolin and consequent blockade of this increase by control compounds was also observed in *Pkd1* knockout cells (mIMRFNPKD 5E4), as illustrated by three independent experiments presented in **Supplemental Figure S5**.

### Identification of Modulators of Cyst Growth

**Figure 3.**
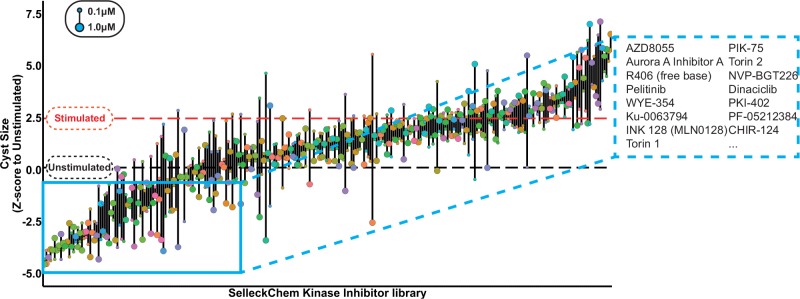
SelleckChem kinase inhibitor library screen. Kinase inhibitors were screened in quadruplicate (technical replicates) at 1 and 0.1 µM, in the presence of 2.5 µM forskolin, to stimulate cyst growth (procedure shown in **Fig. 1**). Data were *z* score normalized to the plate median and subsequently to the unstimulated control median (unstimulated control, black striped line). Cyst growth induction by forskolin is presented by a red striped line. Compound effects are represented by means of quadruplicate wells of two tested concentrations. Top 15 hit molecules are presented in the right cutout. The replicate-adjusted Z′ factor for this primary screen reached −0.93 between stimulated and unstimulated control conditions.

**Figure 4.**
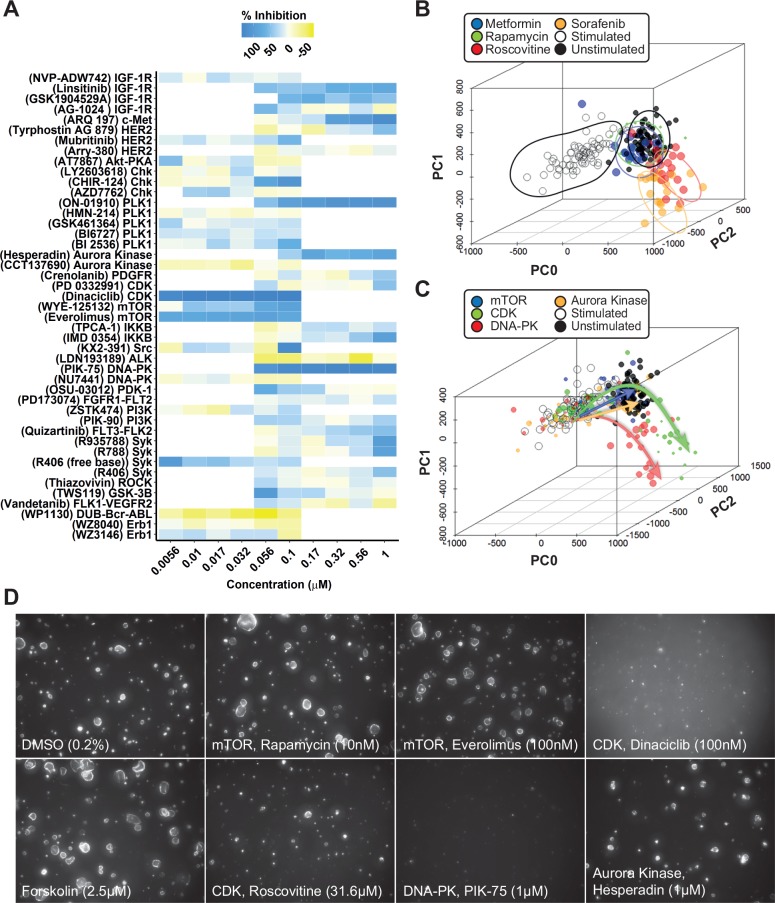
Phenotypic analysis discriminates potentially undesirable compound effects. (**A**) Validation of compounds from each target identified in **Figure 3**. Mean percent inhibition (technical replicates representing triplicate wells) of cyst growth is depicted by a color scale from yellow (no inhibition of forskolin-induced cyst growth) to blue (complete inhibition of forskolin-induced cyst growth). Standard deviations are not included in this plot; for reference purposes, several dose curves are included in **Supplemental Figure S6**. A mean Z′ factor of +0.36 between stimulated and unstimulated control conditions was calculated over six plates. (**B,C**) Multiparametric (PCA) analysis (PCA plot summarizes 84% of variation in the entire dataset) identifies different compound clusters, as shown by the contour plots. Forskolin-stimulated controls (large cysts) are represented as empty circles; unstimulated controls (small cysts) are represented as black dots. Data points represent single wells. (**C**) PCA plot summarizing 84% of variation in the entire dataset. Trajectories of different compound types are indicated by arrows (mTOR inhibitors, blue; CDK inhibitors, green; DNA-PK inhibitors, red; aurora kinase inhibitors, orange). Data points represent single wells; point size correlates with molecule concentration (legend omitted for presentation purposes). (**D**) Representative images from conditions shown as in panels A–C. Novel phenotypes identified in panels B and C are illustrated by images of 100 nM dinaciclib and 1 µM PIK-75.

Having established that inhibitors targeting pathways known to be important in cyst growth can also prevent cyst swelling in our model, we screened a library of 273 kinase inhibitors (SelleckChem-L1200) with described molecular targets. Therefore, in addition to potentially providing therapeutically interesting molecules, the selected hits could also be used to relate compound efficacy to the cellular signaling pathways that may be involved. mIMCD3 sh*Pkd1* cells were cultured in 3D hydrogels in 384-well plates, in which they formed cysts over a period of 72 h. Subsequently, we coexposed cysts with 2.5 µM forskolin and kinase inhibitors from the SelleckChem compound library in quadruplicate wells at 0.1 and 1 µM for 72 h, as illustrated in **Figure 1B**. Cysts were then fixed and stained and imaged using the BD Pathway 855 imager. Image data were processed and quantified with Ominer software and cyst size was *z* score normalized to the unstimulated control median. Screening results are presented in **Figure 3**. Forskolin-induced cyst swelling and various kinase inhibitors inhibited this effect at 0.1 and/or 1 µM (**Fig. 3**). In this initial screen, we calculated an average Z′ factor of −0.93 between unstimulated and stimulated controls. This weak Z′ factor appeared to be largely due to high variation between technical replicates within the stimulated control group, associated with a variable proportion of poorly expanding cysts in each well. We attributed this to suboptimal cell health as a result of the use of cryopreserved cells in the assay, since introducing a 3-day recovery period after thawing of cryopreserved cells improved the Z′ factor to +0.36 in the validation screen. In the primary screen, active molecules were selected based on a reduction of cyst size relative to the stimulated control (red striped line) to a *z* score for a cyst size of ≤0 (equal to or smaller than the unstimulated control median) at either 0.1 or 1 µM. Inhibitors selected using this threshold included many inhibitors targeting mTOR, aurora A kinase, CDK, IGF-1R, and dual mTOR/PI3K inhibitors. For identified active molecules, we obtained their respective molecular target and validated the efficacy only of compounds with the highest affinity for the targets (shown in **Suppl. Table S4**), at six concentrations, decreasing from 1 or 0.1 µM (depending on the efficacy of the molecule in the primary screen) (**Fig. 4A**). For visualization of compound effects as a function of dose, the mean cyst size of quadruplicate wells was scaled to percent inhibition of forskolin-induced cyst swelling, and this inhibition was presented graphically as a heat map from yellow (no inhibition) to dark blue (potent inhibition) (**Fig. 4A**). Our validation screen showed that the activity of most of the active compounds identified in the primary compound screen could be confirmed. Statistical significance was calculated using one-way analysis of variance (ANOVA) and correlated with statistical significance of the primary screen. Statistical significance was found to overlap for 58% of selected molecules (**Suppl. Table S5**). Dose–response curves for several of the compounds presented in **Figure 4A** have been included in **Supplemental Figure S6A–H** to illustrate variation in efficacy between technical replicates. Multi-parametric phenotypic analysis using principal components revealed that different compounds with different molecular targets induced novel phenotypes (**Fig. 4B**). Forskolin (empty circles) induced a phenotypic change characterized by an increase in cyst swelling, which, after PCA, revealed a shift in PC0 and PC2. Coexposure with either rapamycin (green) or metformin (blue) induced a phenotype indistinguishable from unstimulated controls (black). In contrast, exposure of forskolin-stimulated cysts to CDK inhibitor roscovitine (red) or B-Raf/Raf-1 inhibitor sorafenib (orange) caused this phenotypic change to overshoot, causing a novel phenotype (**Fig. 4B,D**). We further observed that while roscovitine and sorafenib inhibited cyst growth similarly to rapamycin and metformin, they also appeared to have an effect on nuclei counts (**Suppl. Fig. S7**), which potentially is an indication of growth inhibition or cytotoxicity related to the concentration of these inhibitors and/or due to their mechanism of action. Using this same projection model that identified the novel phenotypes of roscovitine and sorafenib, we plotted compounds targeting different kinases in the same chart (**Fig. 4C**). Indeed, other CDK inhibitors (green), such as dinaciclib, were found to cluster in the same region of the PCA plot with roscovitine (**Fig. 4B,C** compared). In addition to the phenotype induced by dinaciclib, DNA-PK inhibitor PIK-75 (red) also induced a novel phenotype characterized by low cell count (**Fig. 4C,D**). Although this inhibitor would classify as a potent inhibitor of cyst growth based on cyst size (**Fig. 4A**), this molecule induced a phenotypic change that was very different from that of the unstimulated control (black). The novel phenotypes induced by dinaciclib and PIK-75 may be potentially undesirable and appear to be characterized by a lower number of viable cells, as shown in **Figure 4D**.

### mTOR Inhibitors, but Not PI3K inhibitors, Block Forskolin-Induced Cyst Swelling

**Figure 5.**
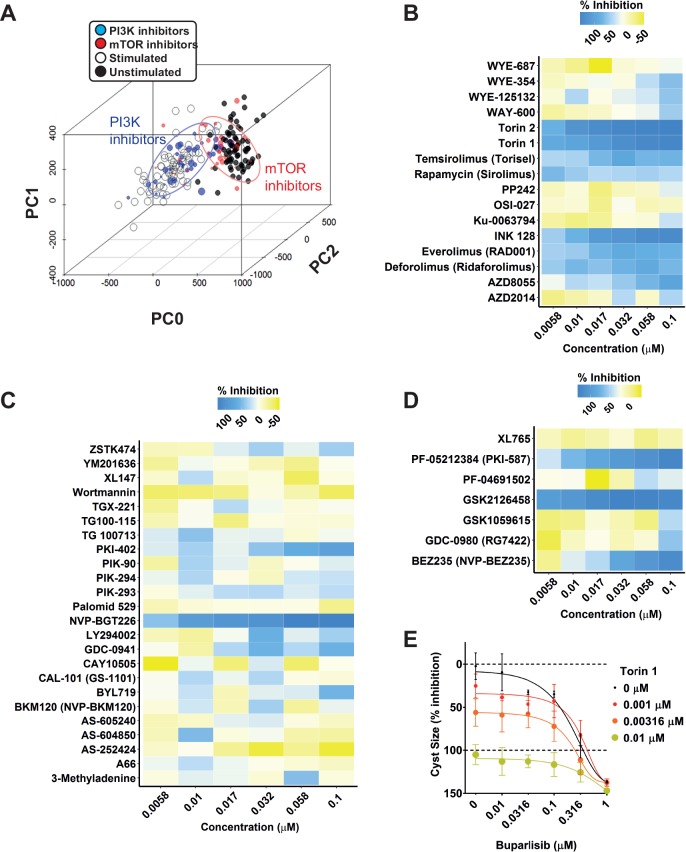
mTOR inhibitors, but not PI3K inhibitors, prevent forskolin-induced cyst swelling. (**A**) PCA plot comprising 84% of variation in the entire dataset. mTOR inhibitors (red circles) cluster together with unstimulated control (black dots), whereas PI3K inhibitors (blue circles) cluster with the stimulated condition (empty circles). Data points represent single wells; inhibitor point size correlates with molecule concentration (legend omitted for presentation purposes). (**B**) Validation of mTOR inhibitors at concentrations of 6–100 nM. Cyst growth inhibition displayed by a color gradient ranging from yellow (no inhibition) to blue (inhibition). The color represents the mean value of triplicate wells (technical replicates; mean Z′ factor over eight plates of 0.28 between stimulated and unstimulated control conditions; also for panels C and D). (**C**) Validation of PI3K inhibitors; color scale as in panel B. (**D**) Validation of dual PI3K/mTOR inhibitors; color scale as in panel B. (**E**) Combination of mTOR inhibitor torin 1 with PI3K inhibitor buparlisib (NVP-BKM120) to assess synergy in mIMRFNPKD 5E4 cells. Values represent mean ± SD of quadruplicate wells (technical replicates; Z′ factor of +0.64 between stimulated and unstimulated control conditions).

Based on our kinase inhibitor screen described in **Figure 3**, we discovered that almost all mTOR inhibitors showed inhibitory activity on cyst growth, but most PI3K inhibitors showed at most only mild inhibition, which was unexpected in view of the expected role of PI3K in PKD.^22–24^ This was confirmed using PCA-based visualization and showed that mTOR inhibitors blocked the forskolin-induced phenotype, whereas PI3K inhibitors had no effect (**Fig. 5A**). We therefore retested a collection of 40 inhibitors that were annotated as mTOR and PI3K inhibitors from the SelleckChem library, and also tested a panel of 7 dual PI3K/mTOR inhibitors at concentrations of up to 0.1 µM (**Fig. 5B–D**). Cyst size was measured and scaled to percent inhibition, denoted by a color scale from yellow (inactive) to dark blue (potent inhibition). Most mTOR inhibitors, and most notably torins 1 and 2, showed potent inhibitory activity (**Fig. 5B**), whereas most PI3K inhibitors had low efficacy (**Fig. 5C**). We noted that the very few PI3K inhibitors that were effective at inhibiting cyst growth may also have activity against mTOR (e.g., NVP-BGT226^25^). Most of the dual PI3K/mTOR inhibitors tested showed potent inhibition of forskolin-induced cyst growth (**Fig. 5D**). We therefore considered whether PI3K, which is aberrantly active in PKD,^22,23^ was not involved in cyst growth in our model, or whether the PI3K inhibition could exert synergistic effects together with mTOR inhibitors. To test this, we selected one mTOR inhibitor, torin 1^26^ that displayed potent activity in our previous screens and has a 1000-fold selectivity for mTOR over PI3K^27^ (**Suppl. Table S4**). Similarly, we selected a PI3K inhibitor, NVP-BKM120 (buparlisib), with reduced potency against mTOR^28^ (**Suppl. Table S4**). **Figure 5E** shows that while both molecules show inhibition of cyst growth at high concentrations, torin 1 is a potent inhibitor at its IC50 concentration, whereas buparlisib shows little activity, except at higher concentrations, when it may also inhibit mTOR signaling.^29^ Furthermore, when combined, these mTOR and PI3K inhibitors did not cause synergistic potentiation of cyst growth inhibition, as illustrated in **Figure 5E** and **Supplemental Figure S8**, upon comparison with the predicted combined response. Based on the performed separate experiments with buparlisib and torin 1, an expected additive effect could be derived (**Suppl. Fig. S8B**). Subsequently, the observed combined effect under combined exposure (e.g., 0.00316 µM torin 1 and 0.1 µM buparlisib) was less than this predicted effect (**Suppl. Fig. S8C**). This discrepancy is reflected by a combination index (*CI*) of >1 (**Suppl. Fig. S8D**). Together, these findings indicate that mTOR, but not PI3K, plays a role in driving forskolin-induced cyst swelling in this model.

## Discussion

To evaluate potential targets and therapeutic modulators of PKD, we developed a fully scalable high-content screening platform that uses 3D cultured cysts to quantify the effects of compounds on cyst growth. We applied this model to screen a kinase inhibitor library (SelleckChem L1200) with predescribed targets. While many small-molecule kinase inhibitors often do not have one single molecular target, but rather target a range of kinases, we used this information to investigate the importance of several signaling cascades in our assay. For example, we identified many mTOR inhibitors among the most active compounds. The importance of mTOR in PKD has been extensively described,^30,31^ so identification of mTOR inhibitors in our selected hits was therefore unsurprising, but confirmative of the validity of the screen. Additionally, we also identified several molecules that targeted CDK, Chk, and aurora A kinase. Since these molecules all target kinases that are important in cell cycle progression, it is possible that these molecules block forskolin-induced cyst swelling by limiting proliferation or other growth-limiting effects that are potentially undesirable from a treatment perspective. Indeed, phenotypic analysis showed that especially CDK (e.g., CDK 1, 2, 5, and 9 inhibitor dinaciclib) and DNA-PK (e.g., DNA-PK and PI3K inhibitor PIK-75) inhibitors induced a novel phenotypic change, characterized by cyst sizes smaller than unstimulated control cysts and loss of cyst integrity, that could indicate a toxic effect, rather than only affecting the size of the cysts. Due to the function of CDKs in regulating the cell cycle and the function of DNA-PK in DNA damage repair,^32^ the identification of these growth inhibitory effects is unsurprising. Hence, these molecules are probably not desirable for prolonged therapeutic use in asymptomatic patients.

Consistent with previous reports,^33^ we identified several inhibitors for IGF-1R with potent inhibitory effects on cyst swelling. Some of these are also reported to have activity against the insulin receptor (IR) at the concentrations that we used, such as linsitinib, which inhibits IGF-1R with an IC50 of 35 nM^34^ and the IR at 75 nM. Additional studies, for example, using function-blocking antibodies, are required to determine whether a combined action on IGR-1R and IR is responsible for the observed effect, rather than sole inhibition of IGF-1R.

The c-Met inhibitor ArQ-197 also showed potent inhibitory activity in our assay, although other c-Met inhibitors, such as PHA-665752 and SGX-523, were inactive in the assay. This suggests that ArQ-197 may induce growth inhibitory effects independently of its effects on the c-Met receptor tyrosine kinase, which is consistent with our previous observations.^15^ Our observation that cyst growth was reduced by IKK-α/β inhibitors suggests an involvement of the NFκB pathway, as previously described.^35^ Additionally, our results hint at the involvement of spleen tyrosine kinase (Syk), which, to our knowledge, has not previously been linked to PKD. Syk may represent an interesting target for various renal diseases.^36^ However, R788 (fostamatinib) and its active metabolite R406 may also inhibit fms-like tyrosine kinase 3 (Flt3) at the tested concentrations, so their inhibitory activity may not be solely due to inhibition of Syk (**Suppl. Table S4**; also quizartinib, an inhibitor of Flt3, displays inhibitory activity as shown in **Fig. 4A**). Interestingly, epidermal growth factor receptor (EGFR) ErbB-1 has previously been described for its involvement in cyst growth;^37^ however, inhibitors for EGFR were not identified as hits in our screen. This may be due to the absence of added EGFR ligands in the 3D cyst cultures. Interestingly, our screen did identify several HER2 (ErbB-2) inhibitors that reduced cyst swelling. HER2 was identified as a potential target in PKD more than 10 years ago, but has received little attention since.^38^

Many mTOR inhibitors were selected as hits, but to our surprise, PI3K inhibitors were not, even though this has been described as a pathway involved in cyst growth.^23,24^ We therefore tested a larger panel of inhibitors for both protein targets and found that indeed, while mTOR inhibitors showed potent efficacy, PI3K inhibitors generally lacked inhibitory activity. We also showed a lack of synergy between PI3K and mTOR inhibitors buparlisib and torin 1, indicating that PI3K does not play a role in cyst growth in this in vitro assay for cyst growth. However, our results do not exclude a role for PI3K in PKD patients, where cyst growth is driven by mutations in the *PKD1* or *PKD2* gene. For instance, PI3K could play a role in different phases of the disease. Furthermore, PI3K may play a more important role in the *PKHD1*-mediated recessive form of PKD.^22^

In conclusion, we have developed a high-throughput-compatible 3D cell culture–based screening platform to identify molecules that affect cystogenesis. We have used this platform to identify molecular targets involved in cyst swelling and have identified several known and novel pathways to be involved in cyst growth in our model. Additionally, we used multiparametric analysis to discriminate compounds that effectively inhibited cyst growth from compounds that inhibited through potentially toxic effects.

## Supplementary Material

Supplementary material
