# Supplementary material for: High-Throughput Phenotypic Screening of Kinase Inhibitors to Identify Drug Targets for Polycystic Kidney Disease
Source: SLAS Discov. 2017 Jun 23;22(8):974–84. doi: 10.1177/2472555217716056 (PMC5574491; doi:10.1177/2472555217716056)
Supplement: Supplementary material [file Booij_et_al_Supplemental_Materials.pdf]

**SUPPLEMENTAL MATERIALS**

# HIGH-THROUGHPUT PHENOTYPIC SCREENING OF KINASE INHIBITORS TO IDENTIFY DRUG TARGETS FOR POLYCYSTIC KIDNEY DISEASE

Tijmen H. Booij[1], Hester Bange[1], Wouter N. Leonhard[2], Kuan Yan[3], Michiel Fokkelman[1], Steven J. Kunnen[2], Johannes G. Dauwerse[2], Yu Qin[1], Bob van de Water[1], Gerard J.P. van Westen[4], Dorien J.M. Peters[3], Leo S. Price[1,3#]

[1]Division of Toxicology, Leiden Academic Centre for Drug Research, Leiden, The Netherlands
[2]Human Genetics, Leiden University Medical Center, Leiden, The Netherlands
[3]OcellO B.V., Leiden, The Netherlands
[4]Division of Medicinal Chemistry, Leiden Academic Centre for Drug Research, Leiden, The Netherlands

#Corresponding author

**Supplemental Table 1: RFNs for Pkd1 exon 15**

| *Sequence Name* | *Target site 1* | *Target site 2* |
|---|---|---|
| *RFNmPKD1ex15g* | AGCAGGATTTCAAAGTGGAC | CTTACTTTCCGACTTCAGGT |

**Supplemental Table 2: Primers flanking RFN target sites**

| *Primer name* | *Primer sequence* |
|---|---|
| **PKD1ex15F2** | CACAGTAGAGGAACCCATTGTGA |
| **PKD1ex15R2** | CACACCACCCAGTTCATTAAAGG |

**Supplemental Table 3: Phenotypic features incorporated in principal component analysis (PCA)**

| *Feature* | *Image Channel* |
|---|---|
| **area (Rhodamine)** | Cytoskeleton |
| **average_length_of_branches (Rhodamine)** | Cytoskeleton |
| **avg_wall_to_outline_center_dist (Rhodamine)** | Cytoskeleton |
| **equivdiameter (Rhodamine)** | Cytoskeleton |
| **feret (Rhodamine)** | Cytoskeleton |
| **major_Axis (Rhodamine)** | Cytoskeleton |
| **maximum_length_of_branches (Rhodamine)** | Cytoskeleton |
| **minFeret (Rhodamine)** | Cytoskeleton |
| **minor_Axis (Rhodamine)** | Cytoskeleton |
| **nNumber_of_connection_points (Rhodamine)** | Cytoskeleton |
| **number_of_branches (Rhodamine)** | Cytoskeleton |
| **number_of_end_points (Rhodamine)** | Cytoskeleton |
| **perimeter (Rhodamine)** | Cytoskeleton |
| **ratio_Area_BoundingBox_Area (Rhodamine)** | Cytoskeleton |
| **wall_count (Rhodamine)** | Cytoskeleton |
| **zernike_order_0_0 (Rhodamine)** | Cytoskeleton |
| **zernike_order_1_1 (Rhodamine)** | Cytoskeleton |
| **zernike_order_2_0 (Rhodamine)** | Cytoskeleton |
| **zernike_order_2_2 (Rhodamine)** | Cytoskeleton |
| **zernike_order_3_3 (Rhodamine)** | Cytoskeleton |
| **zernike_order_4_2 (Rhodamine)** | Cytoskeleton |
| **zernike_order_5_1 (Rhodamine)** | Cytoskeleton |
| **zernike_order_5_3 (Rhodamine)** | Cytoskeleton |
| **zernike_order_5_5 (Rhodamine)** | Cytoskeleton |
| **zernike_order_6_0 (Rhodamine)** | Cytoskeleton |
| **zernike_order_6_2 (Rhodamine)** | Cytoskeleton |
| **zernike_order_6_4 (Rhodamine)** | Cytoskeleton |
| **zernike_order_6_6 (Rhodamine)** | Cytoskeleton |
| **zernike_order_7_1 (Rhodamine)** | Cytoskeleton |
| **zernike_order_7_7 (Rhodamine)** | Cytoskeleton |
| **zernike_order_8_0 (Rhodamine)** | Cytoskeleton |
| **zernike_order_8_2 (Rhodamine)** | Cytoskeleton |
| **zernike_order_8_4 (Rhodamine)** | Cytoskeleton |
| **zernike_order_8_6 (Rhodamine)** | Cytoskeleton |
| **zernike_order_8_8 (Rhodamine)** | Cytoskeleton |
| **zernike_order_9_1 (Rhodamine)** | Cytoskeleton |
| **zernike_order_9_7 (Rhodamine)** | Cytoskeleton |
| **zernike_order_9_9 (Rhodamine)** | Cytoskeleton |
| **avg_child_to_par_center_dist (Hoechst)** | Nucleus |
| **axis_Ratio_Minor_Major (Hoechst)** | Nucleus |
| **child_count (Hoechst)** | Nucleus |
| **eccentricity (Hoechst)** | Nucleus |
| **hu_order_1 (Hoechst)** | Nucleus |
| **roundness (Hoechst)** | Nucleus |
| **zernike_order_2_2 (Hoechst)** | Nucleus |
| **zernike_order_9_3 (Hoechst)** | Nucleus |
| **zernike_order_9_5 (Hoechst)** | Nucleus |

## Supplemental Table 4: Validation screen molecule information

| MOLECULES | TARGET INFORMATION | BRIEF DESCRIPTION (INFORMATION DERIVED FROM WWW.SELLECKCHEM.COM) |
|---|---|---|
| **AG-1024** | IGF1R, IR | Inhibitor of IGF1R/IR autophosphorylation with 7μM and 57μM, respectively. |
| **ARQ 197 (TIVANTINIB)** | c-Met | non-ATP competitive c-Met inhibitor with Ki 0.355μM |
| **ARRY-380** | HER2, EGFR | Inhibitor of HER2 (IC50 8nM) and EGFR (IC50 4μM) |
| **AT7867** | AKT, P70 S6 kinase | IC50 in cell free assays: Akt2 (17nM), PKA (20nM) Akt1 (32nM), Akt3 (47nM), p70 S6K (85nM), RSK1 (>100nM) |
| **AZD7762** | CHK1, CHK2 | IC50 in cell free assays: Chk1 (5nM), Chk2 (<10nM). Less potent against CAM, Yes, Fyn, Lyn, Hck and Lck. |
| **BI 2536** | PLK1 | IC50 in cell free assays: PLK1 (0.83nM), PLK2 (3.5nM), PLK3 (9nM). Molecule also targets PI3K and Met >2μM. |
| **BI6727 (VOLASERTIB)** | PLK1 | IC50 in cell free assays: PLK1 (0.87nM) |
| **BUPARLISIB (NVP-BKM120)** | PI3K (p110α/β/δ/γ) | IC50 in cell free assays: p110α (52nM) p110β (166nM) p110δ (116nM) p110γ (262nM) |
| **CCT137690** | Aurora A, Aurora B, Aurora C | IC50: Aurora A (15nM), Aurora C (19nM), Aurora B (25nM) |
| **CHIR-124** | CHK1 | IC50 in cell free assays: Chk1 (0.3nM), FLT3 (5.8nM), PDGFR (6.6nM), GSK-3 (23.3nM), Fyn (98.8nM). Also targets PKA, PKCγ, LCK, CDK2, VEGFR1, Cdc2, VEGFR2, PKCβ2, PKCα, Chk2 <1uM. |
| **CRENOLANIB (CP-868596)** | PDGFR-α, PDGFRβ | Kd in CHO cells: PDGFRα (2.1nM), PDGFRβ (3.2nM) |
| **DINACICLIB (SCH727965)** | CDK2, CDK5, CDK1, CDK9 | IC50 in cell free assays: CDK2 (1nM), CDK5 (1nM), CDK1 (3nM), CDK9 (4nM). Also blocks thymidine DNA incorporation. |
| **EVEROLIMUS (RAD001)** | mTOR | IC50 in cell free assays: mTOR (FKBP12) (1.6-2.4nM) |
| **GSK1904529A** | IGF1R, IR | IC50 in cell free assays: IR (25nM), IGF1R (27nM), B-Raf (>2μM) and others at higher concentrations. |
| **GSK461364** | PLK1 | Ki in cell free assay: PLK1 (2.2nM) |
| **HESPERADIN** | Aurora B | IC50 in cell free assays TbAUK1 (40nM), Aurora B (250nM) |
| **HMN-214** | PLK1 | Prodrug of HMN-176. Little in vitro data is available but causes cytotoxic effects in nM-range. |
| **IMD 0354** | IKK-β | Affects NFkB nuclear translocation >0.5μM. |
| **KX2-391** | Src | Growth inhibition in cancer cell lines GI 9-60nM. |
| **LDN193189** | ALK2, ALK3 | Inhibits transcriptional activity of ALK2 (5nM) and ALK3 (30nM) receptors in cell-based assays. |
| **LINSITINIB (OSI-906)** | IGF1R, IR | Inhibits IR (75nM) and IGR1R (35nM) in cell-free assays. |
| **LY2603618 (IC-83)** | CHK1 | Inhibits CHK1 (IC50 7nM) in vitro. |
| **MUBRITINIB (TAK 165)** | HER2 | HER2 inhibitor with IC50 of 6nM in BT-474 cells. |
| **NU7441 (KU-57788)** | DNA-PK | Inhibits DNA-PK with IC50 14nM, mTOR at 1.7μM and PI3K at 5μM |
| **NVP-ADW742** | IGF1R | Inhibits IGF1R with IC50 170nM |
| **ON-01910** | PLK1 | Inhibits PLK1 with IC50 9nM in cell-free assays. |
| **OSU-03012** | PDK-1 | IC50 5μM, derivative of celecoxib. |
| **PD 0332991 (PALBOCIC LIB) HCL** | CDK4, CDK6 | IC50 in cell free assays: CDK4/CyclinD3 (9nM), CDK4/CyclinD1 (11nM), CDK6/CyclinD2 (15nM) |
| **PD173074** | FGFR1, VEGFR2 | IC50 in cell free assays: FGFR1 (25nM), VEGFR2 (100-200nM), c-Src (19.8μM) |
| **PIK-75** | DNA-PK, PI3K (p110 α, β, γ and δ) | IC50 in cell free assays: DNA-PK (2nM), p110α (5.8nM), p110β (1.3μM), p110γ (76nM), p110δ (0.51μM) |
| **PIK-90** | PI3K (p110 α, β, γ and δ) | IC50 in cell free assays: p110α (11nM), p110β (350nM), p110γ (18nM), p110δ (58nM) |
| **QUIZARTINIB (AC220)** | FLT3 | IC50 in cell lines: FLT3 (IDT) (1.1nM), FLT3 (WT) (4.2nM). Kd for FLT3 1.6nM. |
| **R406** | Syk | Inhibits Syk with IC50 41nM in cell free assays. Also inhibits FLT3. |
| **R406(FREE BASE)** | Syk | Inhibits Syk with IC50 41nM in cell free assays. Also inhibits FLT3. |
| **R788 (FOSTAMATINIB)** | Syk | Prodrug of R406, Syk inhibitor with IC50 of 41nM |
| **R935788 (FOSTAMATINIB DISODIUM, R788 DISODIUM)** | Syk | Prodrug of R406, Syk inhibitor with IC50 of 41nM |
| **THIAZOVIVIN** | ROCK | Inhibits ROCK in low micromolar range. |
| **TORIN-1** | mTOR (mTORC1/2) | IC50 of 2nM/10nM for mTORC1/2, respectively. 1000-fold selectivity for mTOR over PI3K. |
| **TPCA-1** | IKK-β, IKK-α | In a cell free assay, TPCA-1 inhibits IKK-β with an IC50 of 17.9nM, IC50 against IKK-α 400nM, and JNK3 3600nM. |
| **TWS119** | GSK-3β | IC50 in cell free assays: GSK-3β (30nM) |
| **TYRPHOSTIN AG 879 (AG 879)** | HER2 | Inhibits HER2 with an IC50 of 1.0μM |
| **VANDETANIB (ZACTIMA)** | VEGFR, EGFR | Inhibits VEGFR2 in cell free assays with an IC50 of 40nM. Also inhibits VEGFR3 and EGFR with IC50 110nM and 500nM. |
| **WP1130** | Deubiquitinase (USP5, UCH-L1, USP9x, USP14, and UCH37) and Bcr/Abl, JAK2, STAT | Degrasyn is a deubiquitinase that also inhibits Bcr/Abl at 1.8μM |
| **WYE-125132** | mTOR | Potent mTOR inhibitor with an IC50 of 0.19nM. Highly selective over PI3Ks. |
| **WZ3146** | EGFR | Mutant-selective EGFR inhibitor with IC50 2-14nM. <100-fold less potent against WT EGFR. |
| **WZ8040** | EGFR | Mutant-selective irreversible EGFR inhibitor. <100 fold less potent against WT EGFR |
| **ZSTK474** | PI3K (p110 α, β, γ and δ) | IC50 in cell free assays: p110α (16nM), p110β (44nM), p110γ (49nM), p110δ (4.6nM) |

**Supplemental Table 5: Statistical significance of selected hits**

| COMPOUND | Conc. (µM) | ONE WAY ANOVA WITH DUNNETT'S MULTIPLE COMPARISON TEST (P<0.05)* | | Correlates |
|---|---|---|---|---|
| | | Primary Screen | Validation Screen | |
| AG-1024 | 1 | 0.9995 | 0.9915 | Yes |
| ARQ 197 | 1 | 0.0004 | 0.0001 | Yes |
| ARRY-380 | 1 | 0.999 | 0.9991 | Yes |
| AT7867 | 0.1 | 0.1666 | 0.9913 | Yes |
| AZD7762 | 0.1 | 0.0004 | 0.9996 | No |
| BI 2536 | 0.1 | 0.0001 | 0.0001 | Yes |
| BI6727 | 0.1 | 0.0458 | 0.9803 | No |
| CCT137690 | 0.1 | 0.0001 | 0.9986 | No |
| CHIR-124 | 0.1 | 0.0001 | 0.0001 | Yes |
| CRENOLANIB | 1 | 0.0008 | 0.0686 | No |
| DINACICLIB | 0.1 | 0.0001 | 0.0001 | Yes |
| EVEROLIMUS | 0.1 | 0.0001 | 0.0001 | Yes |
| GSK1904529A | 1 | 0.4917 | 0.0147 | No |
| GSK461364 | 0.1 | 0.0058 | 0.898 | No |
| HESPERADIN | 1 | 0.0001 | 0.0001 | Yes |
| HMN-214 | 0.1 | 0.0001 | 0.9995 | No |
| IMD 0354 | 1 | 0.0001 | 0.0001 | Yes |
| KX2-391 | 0.1 | 0.0057 | 0.0001 | Yes |
| LDN193189 | 1 | 0.0001 | 0.9991 | No |
| LINSITINIB | 1 | 0.0001 | 0.0001 | Yes |
| LY2603618 | 0.1 | 0.0011 | 0.9991 | No |
| MUBRITINIB | 0.1 | 0.024 | 0.0007 | Yes |
| NU7441 | 0.1 | 0.0002 | 0.9835 | No |
| NVP-ADW742 | 0.1 | 0.0001 | 0.9985 | No |
| ON-01910 | 1 | 0.0001 | 0.0001 | Yes |
| OSU-03012 | 1 | 0.0001 | 0.9997 | No |
| PD 0332991 | 1 | 0.0001 | 0.0145 | Yes |
| PD173074 | 1 | 0.9995 | 0.87 | Yes |
| PIK-75 | 1 | 0.0001 | 0.0001 | Yes |
| PIK-90 | 1 | 0.1057 | 0.1812 | Yes |
| QUIZARTINIB | 1 | 0.0186 | 0.0086 | Yes |
| R406 | 1 | 0.0067 | 0.0125 | Yes |
| R406 (FREE BASE) | 0.1 | 0.0001 | 0.3736 | No |
| R788 | 1 | 0.0193 | 0.0001 | Yes |
| R935788 | 1 | 0.3575 | 0.0001 | No |
| THIAZOVIVIN | 1 | 0.603 | 0.9994 | Yes |
| TPCA-1 | 1 | 0.0002 | 0.8242 | No |
| TWS119 | 1 | 0.9808 | 0.9996 | Yes |
| TYRPHOSTIN AG 879 | 1 | 0.9981 | 0.007 | No |
| VANDETANIB | 1 | 0.1431 | 0.3565 | Yes |
| WP1130 | 1 | 0.0001 | 0.0943 | Yes |
| WYE-125132 | 0.1 | 0.0001 | 0.0001 | Yes |
| WZ3146 | 0.1 | 0.0001 | 0.592 | No |
| WZ8040 | 0.1 | 0.0001 | 0.6997 | No |
| ZSTK474 | 1 | 0.0001 | 0.4663 | No |

***Statistical significance was assessed compared to forskolin-stimulated controls using ordinary one-way ANOVA with Dunnett’s multiple comparison test. Differences were considere significantly different for adjusted P<0.05.**

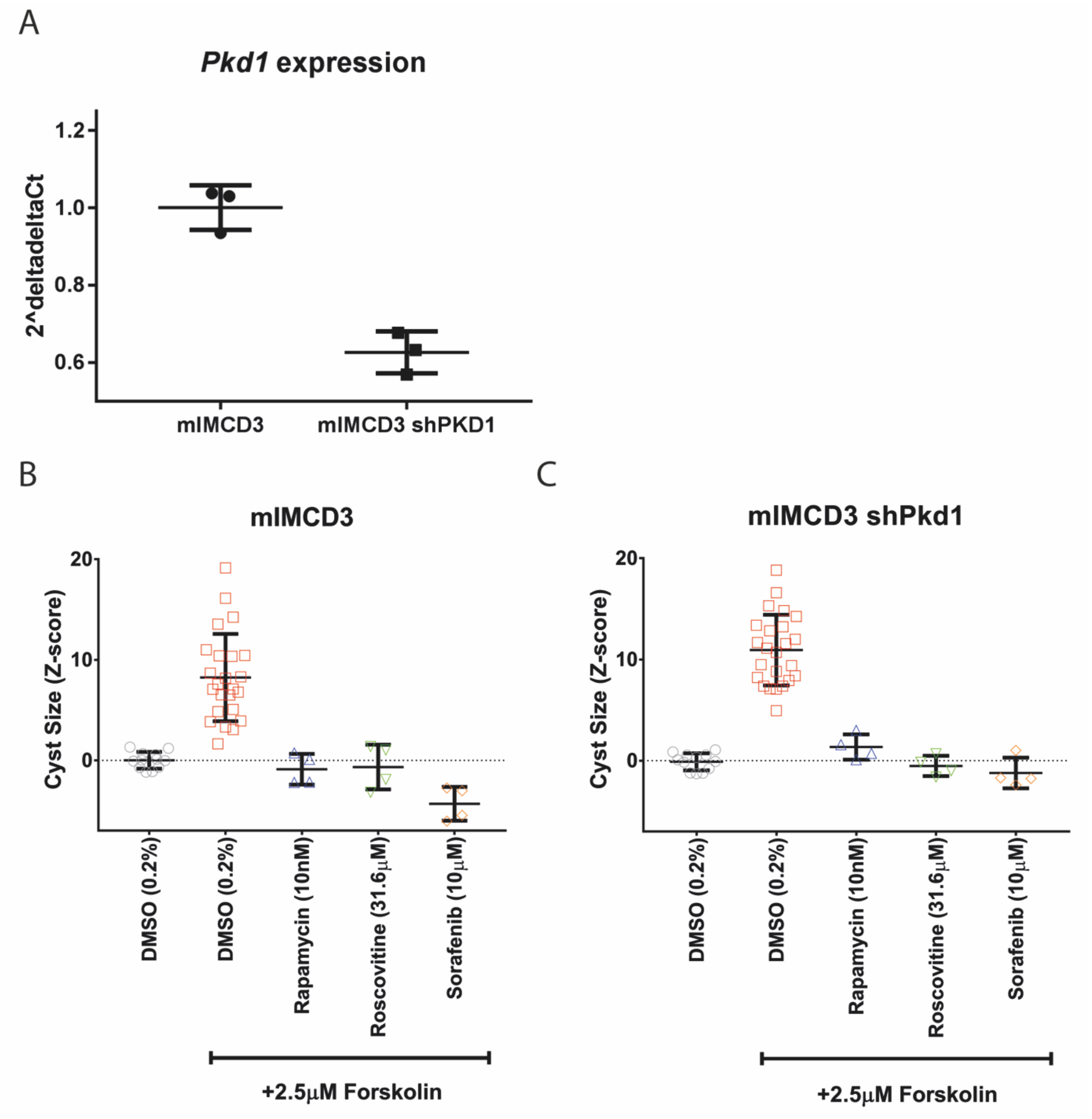


**Supplemental Figure 1**

Pkd1 expression is reduced in mIMCD3 shPkd1 cells. A) qPCR showing reduced expression of *Pkd1* using the $2^{\Delta\Delta Ct}$ method (means ± SD of triplicates). qPCR was performed three times. B and C) Control conditions in both mIMCD3 wt and mIMCD3 shPkd1 cells. Cyst growth depicted by cyst size *z*-scored to unstimulated control (DMSO 0.2%, grey circles) median (dotted black line at $y$=0). Induction of cyst growth by forskolin demonstrated by red squares. Individual datapoints and mean ± SD shown.

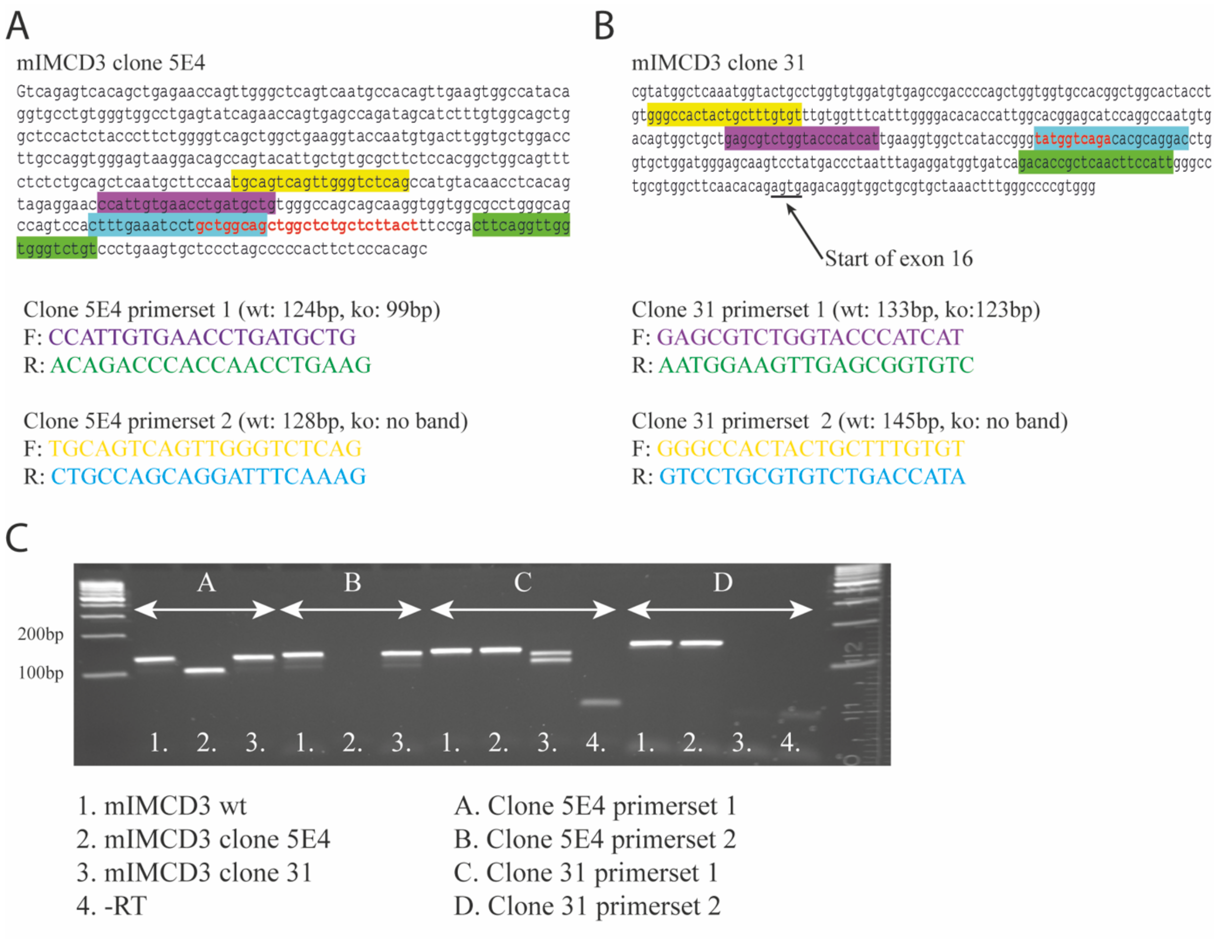


**Supplemental Figure 2**

Generation of *Pkd1* knockout cell lines. A and B) primer sets for both 5E4 and 31 clones for PCR depicted in C. C) PCR revealing deletion in *Pkd1* gene in clone 5E4 and clone 31. Clone 5E4 was selected due to improved growth characteristics.

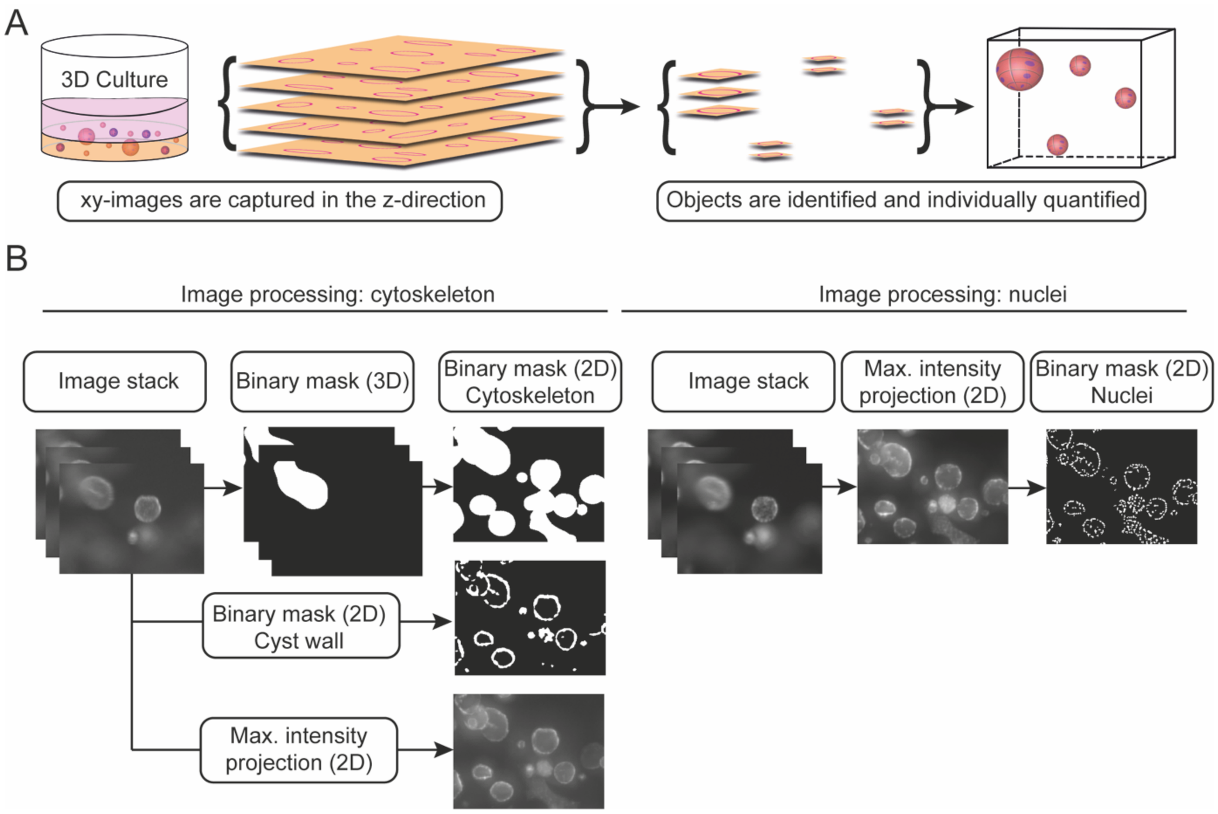


**Supplemental Figure 3**

Schematic representation of image analysis procedure. A) Images are captured at 50μm intervals throughout the *z*-axis of the gel and objects are extracted from each image plane and individually quantified. B) Image stacks (cutouts shown) of approximately 25 images per well from both cytoskeleton and nucleus image channels are processed to binary masks for quantification of phenotypic descriptors.

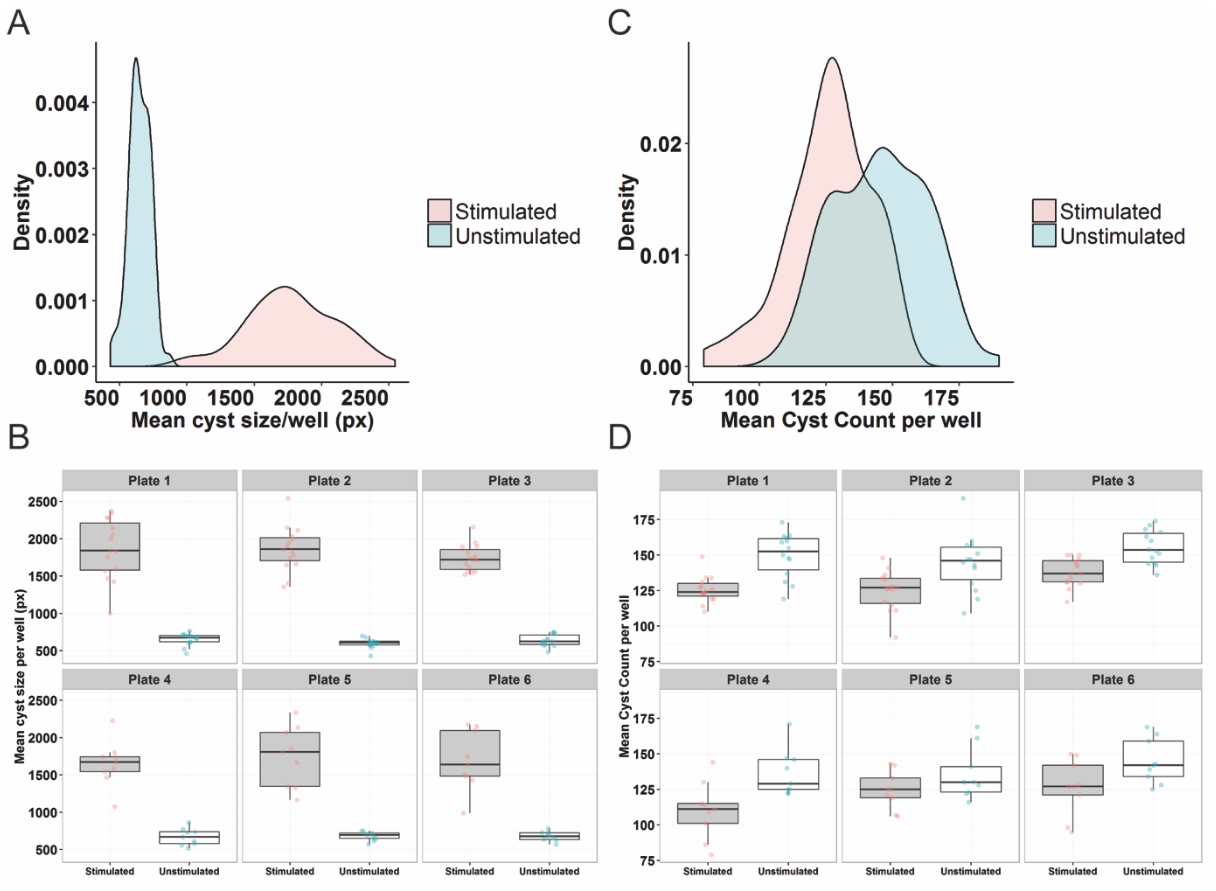


**Supplemental Figure 4**

Forskolin-treatment increases cyst growth. A) Density plot of validation screen showing non-normalized cyst size from all six plates in the validation for unstimulated (0.2% DMSO) and stimulated (2.5µM Forksolin) conditions. B) Increased cyst growth after forskolin treatment is highly similar over different plates. C) As in A, but showing the number of cysts per well. D) As in B, number of cysts per well is similar among the six plates.

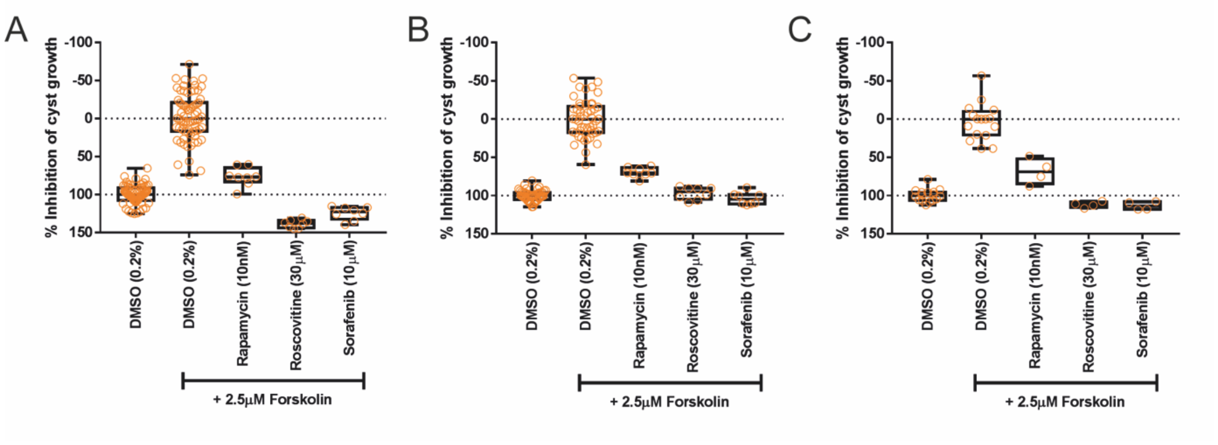


**Supplemental Figure 5**

Forskolin-induced mIMRFNPKD 5E4 cyst growth is inhibited by rapamycin, roscovitine and sorafenib in three independent experiments (A,B,C). Individual wells shown as orange circles. Box plot whiskers represent min to max.

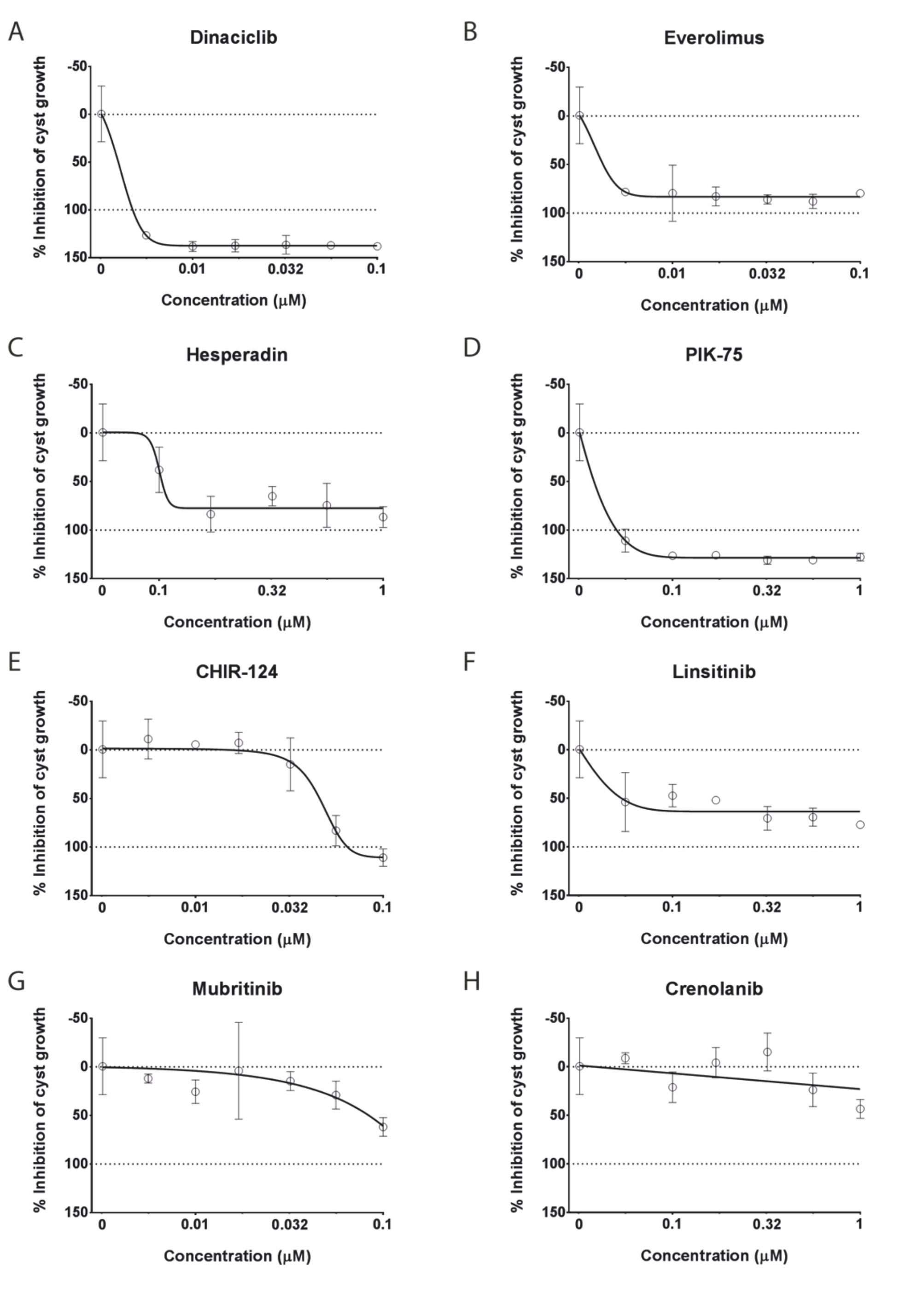


**Supplemental figure 6**

Concentration- cyst growth inhibition curves of data presented in main figure 4A. Black circles represent mean values of three wells (technical replicates) and error bars represent SD to illustrate intra-experimental variation. In case when error bars are not visible, the SD is smaller than the symbol height. A) Dinaciclib, B) Everolimus, C) Hesperadin, D) PIK-75, E) CHIR-124, F) Linsitinib, G) Mubritinib, H) Crenolanib.

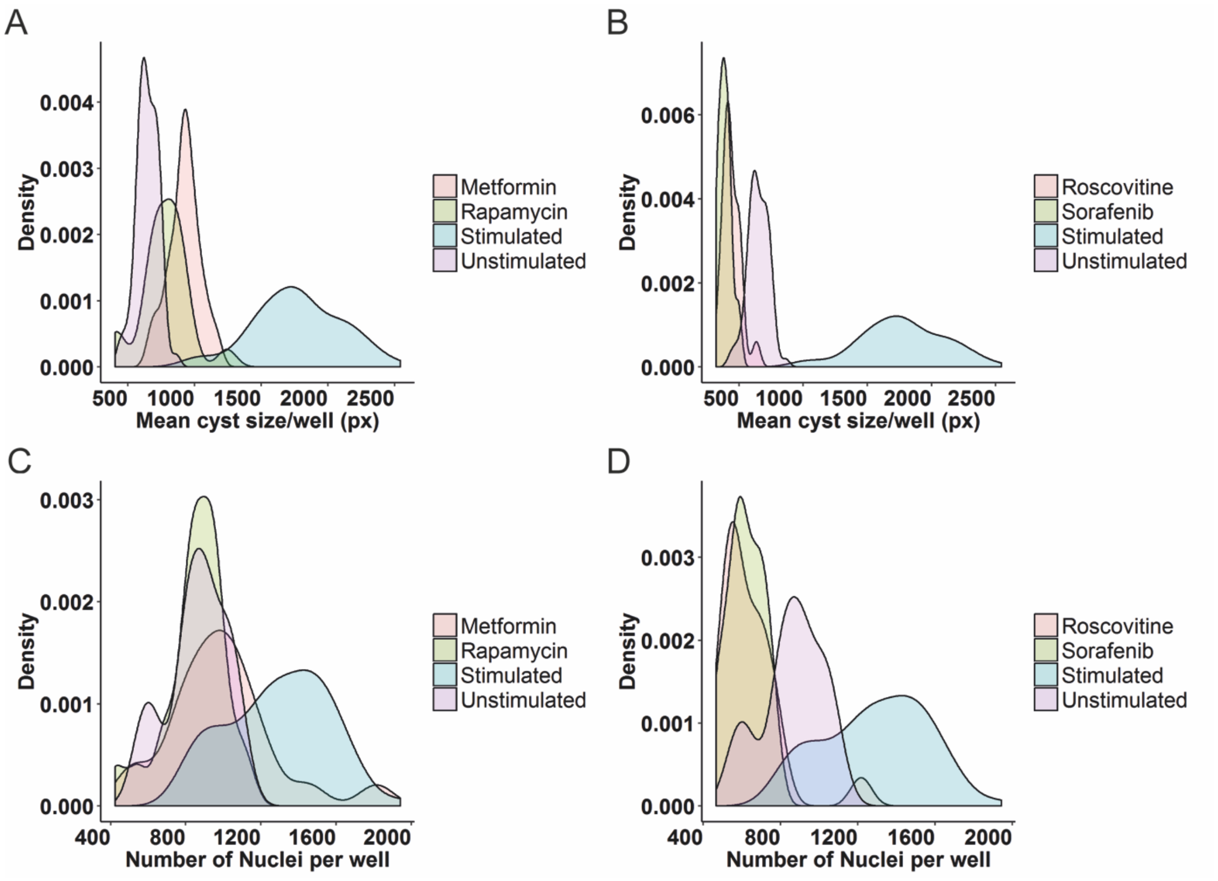


**Supplemental figure 7**

Roscovitine and sorafenib may affect cyst phenotype by reducing cell number. This data is derived from the validation screen as depicted in figure 4 and supplemental figure 4. A) Density plot as in supplemental figure 4 showing that 5mM metformin and 10nM rapamycin (pink and yellow) slow forskolin-induced cyst growth compared to stimulated (2.5µM forskolin, blue) condition. B) Roscovitine (31.6µM, pink) and sorafenib (10µM, yellow) slow forskolin-induced cyst growth compared to stimulated (2.5µM forskolin, blue) condition. C) Metformin and rapamycin do not cause a reduction in the number of nuclei (as derived from a maximum intensity projection of a 3D image stack) relative to unstimulated (0.2% DMSO vehicle, purple) control. D) Roscovitine (pink) and sorafenib (yellow) lower the number of nuclei (as derived from a maximum intensity projection of a 3D image stack) relative to the unstimulated condition (0.2% DMSO vehicle, purple).

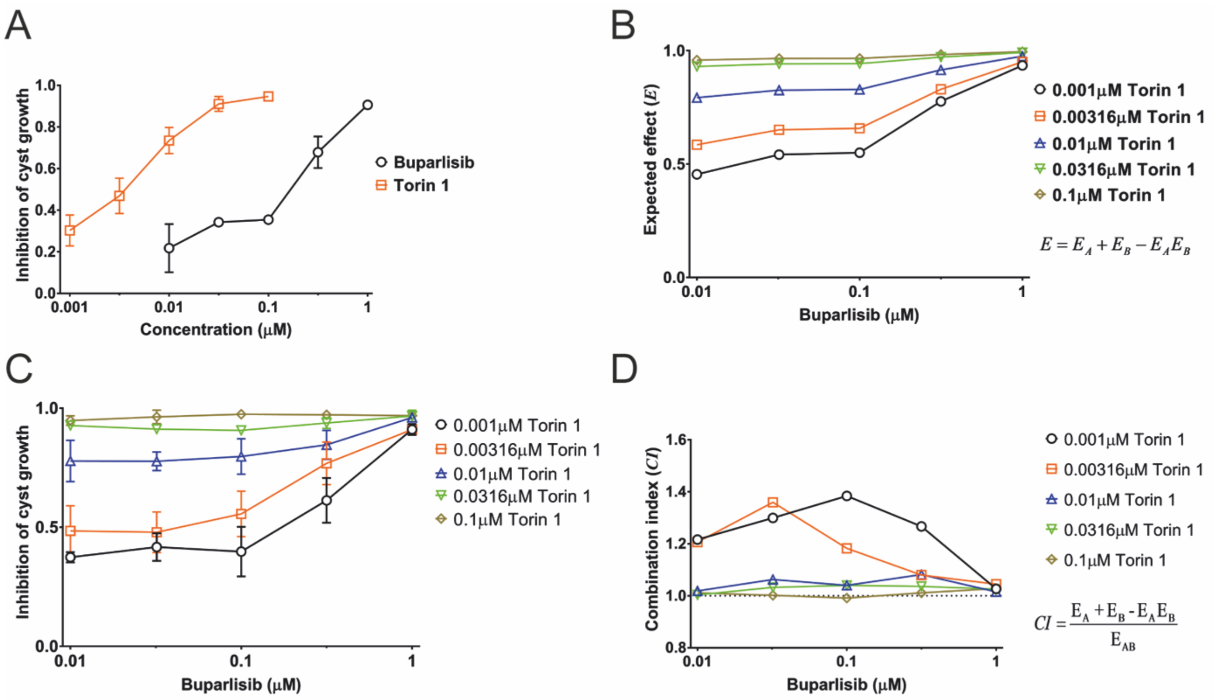


**Supplemental Figure 8**

Assessment of synergistic effects of mTOR inhibitor torin 1 and PI3K inhibitor buparlisib (NVP-BKM-120) in mIMRFNPKD 5E4 cells. When combined, these inhibitors do not cause synergistic potentiation of cyst growth inhibition. A) Experimental measurement of inhibition of forskolin-induced cyst growth by buparlisib and torin 1 scaled between 0 (no inhibition) and 1 (max inhibition). Data presented represent means ± SD of four wells (technical replicates). In case when error bars are not visible, the SD is smaller than the symbol height. B) Predicted inhibitory activity of combinations of torin 1 and buparlisib, scaled between 0 (no inhibition) and 1 (max inhibition). This prediction used the formula $E=E_A+E_B-E_AE_B$ where $E_A$ is the effect of torin 1 and $E_B$ is the effect of buparlisib C) Experimental measurement cyst growth inhibition by combinations of buparlisib and torin 1. Response scaled between 0 (no inhibition) and 1 (max inhibition). Data presented represent means ± SD of four wells (technical replicates). In case when error bars are not visible, the SD is smaller than the symbol height. D) Combination index *CI* was defined by dividing the predicted response *E* with the observed response of combinatory therapy $E_{AB}$. A $CI<1$ indicates synergistic effects, a $CI>1$ indicates antagonistic effects, in contrast to an expected additive effect when $CI=1$. The observed effect of some conditions (e.g. 0.00316μM torin 1 and 0.1μM buparlisib) in C was mildly less potent than the effects predicted in B, causing $CI>1$. For other combinations, *CI* was 1, indicating only additive effects.
